# Exploration of predictive biomarkers for postoperative recurrence of stage II/III colorectal cancer using genomic sequencing

**DOI:** 10.1002/cam4.4710

**Published:** 2022-03-28

**Authors:** Fumishi Kishigami, Yosuke Tanaka, Yoko Yamamoto, Toshihide Ueno, Shinya Kojima, Kazuhito Sato, Satoshi Inoue, Saori Sugaya, Soichiro Ishihara, Hiroyuki Mano, Masahito Kawazu

**Affiliations:** ^1^ Division of Cellular Signaling National Cancer Center Research Institute Tokyo Japan; ^2^ Department of Surgical Oncology The University of Tokyo Tokyo Japan

**Keywords:** colorectal cancer, precision medicine, whole genome sequence, ZFP36L2

## Abstract

Postoperative recurrence of colorectal cancer (CRC) eventually leads to therapeutic failure; therefore, treatment strategies based on accurate prediction of recurrence are urgently required. To identify biomarkers that can predict treatment outcomes, we compared the mutational profiles of surgically resected specimens from patients with recurrent cancer with those from patients with non‐recurrent cancer. Target sequencing, whole‐exome sequencing (WES), or whole‐genome sequencing (WGS) was performed on 89 and 58 tumors from recurrent and non‐recurrent cases, respectively. WGS revealed the driver mutations that were not detected with target sequencing or WES, including the structural variations affecting *ZFP36L2*. Loss of function of *ZFP36L2* was frequently observed in primary tumors from recurrent cases. Furthermore, the recurrence‐free survival of patients with loss of function of *ZFP36L2* was significantly shorter relative to patients with no loss of *ZFP36L2* function. In summary, the study demonstrated that detailed genomic analysis could help improve precision medicine for CRC.

## Graphical abstract

Loss of function of ZFP36L2 was frequently observed in recurrent colorectal cancers. Furthermore, the recurrence‐free survival of colorectal cancer patients with loss of function of ZFP36L2 was significantly shorter relative to patients with no loss of ZFP36L2 function.

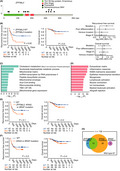

## 1 INTRODUCTION

Colorectal cancer (CRC) is the second leading cause of cancer‐related deaths in Japan (Vital Statistics Japan, Ministry of Health, Labor and Welfare, https://www.mhlw.go.jp/english/database/db‐hw/vs01.html). While radical resection is the first line of treatment, postoperative adjuvant chemotherapy is usually administered to patients with stage III CRC. Although the postoperative recurrence rate of stage II CRC is approximately 15%, administration of chemotherapy in such cases is generally left to the discretion of the clinician, since its efficacy for stage II CRC is still unknown.^1^, ^2^, ^3^

Extensive research has elucidated the landscape of genomic aberrations in CRC,^4^, ^5^, ^6^, ^7^ enabling the implementation of precision medicine through therapeutic stratification; however, the application of prognostic prediction based on mutational profiles remains limited. For instance, low expression of *CDX2* is a predictive marker for chemosensitivity,^8^ mutations in *PTPRT* serve as a potential predictive marker for metastatic CRC,^7^ mutations in *BRAF* V600E is a prognostic factor for stage II microsatellite stable (MSS) CRC,^9^ and mutations in *KRAS* or *BRAF* are associated with worse outcomes for patients with stage III MSS CRC.^10^, ^11^ However, the prediction of postoperative recurrence is unsatisfactory. Many challenges remain in improving treatment strategies based on such prediction.

In this study, we aimed to identify additional biomarkers by comparing the mutational profiles of surgically resected specimens from patients with recurrent cancer relative to those from patients with non‐recurrent cancer. Our findings could help improve the prediction of treatment outcomes in CRC and increase the efficacy of therapeutic strategies.

## 2 MATERIALS AND METHODS

### 2.1 Clinical specimens

Tissues containing tumor and corresponding normal mucosa were collected from surgically resected specimens obtained from patients treated at The University of Tokyo Hospital between 2006 and 2019. Specimens were either snap‐frozen in liquid nitrogen immediately after resection and stored at −80°C or immersed in RNAlater in TissueProtect Tubes (Qiagen) overnight at 4°C, and stored at −20°C until use. Genomic DNA was extracted from tissue samples using the DNeasy Blood & Tissue Kit or the QIAamp DNA Mini Kit (Qiagen). Total RNA was extracted with RNA‐Bee (Tel‐Test) and treated with DNase I (Qiagen) using the RNeasy Mini Kit (Qiagen).

### 2.2 Target sequencing

Two hundred and fifty ng genomic DNA from each sample was fragmented to 200‐bp lengths using the LE220 Focused ultrasonicator (Covaris). The End Prep Enzyme Mix (New England Biolabs) was used for fragment‐end repair and the NEBNext Adaptor for Illumina (New England Biolabs) was used for adaptor ligation. Adaptor‐ligated DNA fragments were amplified for nine cycles. Seven hundred and fifty ng of each PCR product was subjected to target capture with custom‐made probes, as described previously.^12^ The captured library was amplified over 11 cycles, and massive parallel sequencing of the isolated fragments was performed with the HiSeq2500 (Illumina,) using the paired‐end option.

### 2.3 Whole‐exome sequencing

Two hundred and fifty ng of genomic DNA from each sample was fragmented to 200‐bp lengths using the Focused ultrasonicator (Covaris). Adaptor‐ligated DNA fragments, obtained as described in the previous sub‐section, were amplified over nine cycles, and 750 ng of each PCR product was used to capture the exonic region using the SureSelect All Exon Kit 6 (Agilent Technologies) and the Sciclone G3 automation system (PerkinElmer). The captured library was amplified over 11 cycles, and massive parallel sequencing of isolated fragments was performed with the HiSeq2500 (Illumina) or the NovaSeq6000 (Illumina).

### 2.4 Whole‐genome sequencing

Five hundred ng of genomic DNA from each sample was used for library preparation using the automated library preparation run on Bravo (Agilent Technologies) with the TruSeq DNA PCR‐Free Library Prep Kit (Illumina). IDT for Illumina‐TruSeq UD Indexes (Illumina) was used as an unrelated adapter for index reads. Massive parallel sequencing of the isolated fragments was performed with the NovaSeq6000 (Illumina).

### 2.5 Sequencing analysis

Reads from target sequencing, paired‐end WES, and WGS were independently aligned to the human reference genome (hg38) using the Burrows‐Wheeler Aligner (http://bio‐bwa.sourceforge.net/) and Bowtie 2 (http://bowtie‐bio.sourceforge.net/bowtie2/index.shtml). Somatic (synonymous and non‐synonymous) mutations were called using an in‐house caller and two publicly available mutation callers: Genome Analysis Toolkit (https://gatk.broadinstitute.org/hc/en‐us) MuTect 2 (https://gatk.broadinstitute.org/hc/en‐us/articles/360037593851‐Mutect2) and VarScan 2 (http://varscan.sourceforge.net/). Mutations were discarded if any of the following criteria were met: total read number < 20, variant allele frequency in tumor samples <0.05, mutant read number in the germline control samples >2, a mutation in only one genome strand, a variant present in normal human genomes of the 1000 Genomes Project dataset (https://www.internationalgenome.org/) or the in‐house database. Gene mutations were annotated using SnpEff (http://snpeff.sourceforge.net). The copy number status was analyzed using an in‐house pipeline that determined the logR ratio (LRR) as follows: (I) homozygous (variant allele frequency [VAF] ≤ 0.05 or ≥ 0.95) or heterozygous (VAF 0.4–0.6) SNPs were selected from the genomes of the related normal samples in the 1000 Genomes Project database; (II) normal and tumor read depths for the selected SNPs were adjusted based on the G + C percentage of a 100‐bp window flanking position; (III) LRR was calculated as $\log_{2}\frac{t_{i}}{n_{i}}$, where $n_{i}$ and $t_{i}$ are normal and tumor‐adjusted depths at position i, respectively; and (IV) each representative LRR was determined by using the median of a moving window (1 Mb) centered at position i. The LRR of the copy number for both alleles, i.e., the major and the minor alleles were determined for every region of the genome. The *p* values for copy number gain or loss of the respective genomic regions were determined from LRRs with a permutation test (100,000 iterations) in GISTIC2. (https://software.broadinstitute.org/cancer/cga/gistic). *Q* values were calculated from the *P* values using the ‘qvalue’ package in R (http://github.com/jdstorey/qvalue). The allele‐specific copy number status was inferred using the LRR in FACETS.^13^ Structural variations (SVs) were detected by analyzing WGS data with the Genomon 2.6.3 pipeline (https://github.com/Genomon‐Project).

### 2.6 Transcriptome sequencing

One μg RNA, extracted from clinical samples, was subjected to mRNA enrichment using the Oligo d(T)25 Magnetic Beads (New England Biolabs). Sequencing libraries were prepared using the NEBNext Ultra Directional RNA Library Prep Kit (New England Biolabs) and sequenced over 120 bp from both ends using the HiSeq2500 (Illumina). The expression level of each gene was calculated using DESeq2 (http://bioconductor.org/packages/release/bioc/html/DESeq2.html) with VST transformation. Gene fusions were detected using DeFuse (https://bitbucket.org/dranew/defuse) and STAR (https://github.com/alexdobin/STAR). Consensus molecular subtype (CMS)^14^ category was determined using RNA‐seq data.

### 2.7 Signature analysis

Mutational signatures were analyzed using the Wellcome Trust Sanger Institute Mutational Signature Framework (http://jp.mathworks.com/matlabcentral/fileexchange/38724‐wtsi‐mutational‐signature‐framework). The optimal number of signatures was determined based on the signature stabilities and average Frobenius reconstruction errors.

### 2.8 Pathway analysis

Differentially expressed genes (DEGs) between the groups were selected using DESeq2 and subjected to pathway analysis using Metascape.^15^ Pathways defined by Gene Ontology (http://www.geneontology.org), Kyoto Encyclopedia for Genes and Genomes (http://www.genome.jp/kegg/), hallmark gene sets, canonical pathways gene sets, BioCarta gene sets (https://cgap.nci.nih.gov/Pathways/BioCarta_Pathways), and the Reactome pathway database (http://www.reactome.org) were used for the analysis. The signature module of “oncogenic signatures” was also included in the study.

### 2.9 Clinical information and survival analysis

Clinical information, including sex, age, preoperative diagnosis, site of the lesion, the experience of preoperative chemoradiotherapy, pathological findings, recurrence, overall survival, and recurrence‐free survival, were obtained from all patients included in the study. Clinical data were anonymized at the Department of Healthcare Information Management, The University of Tokyo.

Patients were assessed after operation at every 3 months, for as long as possible. Patients underwent physical examination, review of symptoms suggestive of recurrence, semi‐yearly computed tomography of chest, abdomen, and pelvis, measuring carcinoembryonic antigen and carbohydrate antigen 19–9 at each visit, and colonoscopy at years 1, 2, 3, 4 and 5. RFS is determined at the first event of recurrent disease (local, distant or regional). OS is determined at the time of decease. Patients without a recurrence or decease were censored at their last assessment for follow‐up.

### 2.10 Clonal analysis

The cellular prevalence of clones carrying individual non‐synonymous mutations was determined using PyClone.^16^ The determined cellular prevalence was used as the input data for LiCHeE^17^ to identify phylogenetic relationships between the clones. Clinicians estimated the mode of recurrence by considering the location of the lesion, the time elapsed between the first and second surgeries, and the surgical procedure. Concordance between the results of clonal analysis and inference based on clinical information was evaluated.

### 2.11 Statistics

Numerical variables were summarized by the median and range. Comparisons of numerical variables between groups were performed using the two‐tailed Wilcoxon rank‐sum test. Comparisons of categorical variables between groups were performed using Fisher's exact test. The survival curve was established by the Kaplan–Meier method and compared using the log‐rank test with Bonferroni adjustment. The Cox proportional‐hazards model was also used to evaluate the effects of multiple variables. Statistical analysis was performed using R 3.5.1 (R Core Team, 2015) or SciPy 1.1.0 (Community Library Project, 2021).

## 3 RESULTS

### 3.1 Overview of patients

**FIGURE 1:**
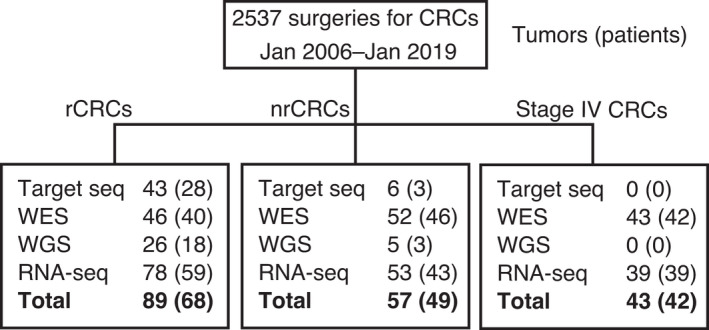
Study overview. Numbers outside parenthesis: tumor specimens subjected to genomic analysis; Numbers inside parenthesis: patients from which tumor specimens were collected; CRC, colorectal cancer; nrCRC, non‐recurrent CRC; rCRC, recurrent CRC; RNA‐seq, transcriptome sequencing; Target seq, target sequencing

From 2537 colorectal cancer (CRC) specimens surgically resected in the University of Tokyo hospital between January 5, 2006, and January 31, 2019, recurrent CRCs (rCRCs) and stage IV CRCs for which fresh frozen tumor tissue was available were selected for this study (Figure 1); 89 tumors from 68 patients that showed recurrence within 5 years after radical surgery, were selected as rCRCs. As a control group, 58 tumors from 49 patients, who remained free from recurrence for at least 5 years, were selected as non‐recurrent CRCs (nrCRCs). The nrCRCs were chosen in order from newest to oldest, so that they would have the same number as the rCRCs. Three samples (one tumor with high microsatellite instability, one associated with Lynch syndrome, and one associated with ulcerative colitis) were excluded from the analysis. In addition, we included 43 tumor tissues from 42 patients with stage IV CRC at the pre‐operative diagnosis. In total, we analyzed 190 samples from 159 patients. Multiple samples were collected from 22 patients (52 samples).

Patients' characteristics are presented in Table 1; the mean age at surgery was 63 years, and 50.9% of the patients were male. The median follow‐up period was 1811 days (67–4620 days), whereas the median period for recurrence after surgery was 906 days (44–2537 days). Compared to those in the cohort from The Cancer Genome Atlas (TCGA) program, the clinical features in our study did not show any difference.

**TABLE 1:** Patient characteristics

Characteristics	rCRC	nrCRC	Stage IV
Age at operation, mean ± standard deviation	64 ± 10	66 ± 12	59 ± 11
Male, number (%)	36 (52.9)	28 (57.1)	17 (40.5)
Site of tumor, number (%)			
Right side colon	15 (22.1)	8 (16.3)	18 (42.9)
Left side colon	28 (41.2)	21 (42.9)	12 (28.6)
Rectum	25 (36.8)	20 (40.8)	12 (28.6)
Invasion depth, number (%)			
T0‐2 (up to SM)	6 (8.82)	8 (16.3)	2 (4.76)
T3 (SS or A)	30 (44.1)	27 (55.1)	13 (31.0)
T4 (SE, SI or AI)	32 (47.1)	14 (28.6)	27 (64.3)
Regional lymph nodes metastasis, number (%)			
N0	37 (54.4)	28 (57.1)	8 (19.0)
N1	22 (32.4)	14 (28.6)	17 (40.5)
N2 or 3	9 (13.2)	7 (14.3)	17 (40.5)
Stage, number (%)			
II	38 (55.9)	28 (57.1)	0 (0.00)
III	30 (44.1)	21 (42.9)	0 (0.00)
IV	0 (0.00)	0 (0.00)	42 (100)
Survival, number (%)			
Dead	22 (32.4)	0 (0.00)	20 (47.6)
Alive	46 (67.6)	49 (100)	22 (52.4)
Total, number	68	49	42

### 3.2 Genomic analysis of rCRC

Target sequencing was conducted using 43 tumors from 28 patients with rCRC and six tumors from three patients with nrCRC. We selected 26 tumors from 18 patients with rCRC and five tumors from three patients with nrCRC, based on the relatively higher tumor content estimated from target sequencing, for analysis via WGS. We selected 46 tumors from 40 patients with rCRC, 52 tumors from 46 patients with nrCRC, and 43 tumors from 42 patients with stage IV CRC for analysis via WES, and 78 tumors from 59 patients with rCRC, 53 tumors from 43 patients with nrCRC, and 39 tumors from 39 patients with stage IV CRC for analysis via transcriptome sequencing (Figure 1).

**FIGURE 2:**
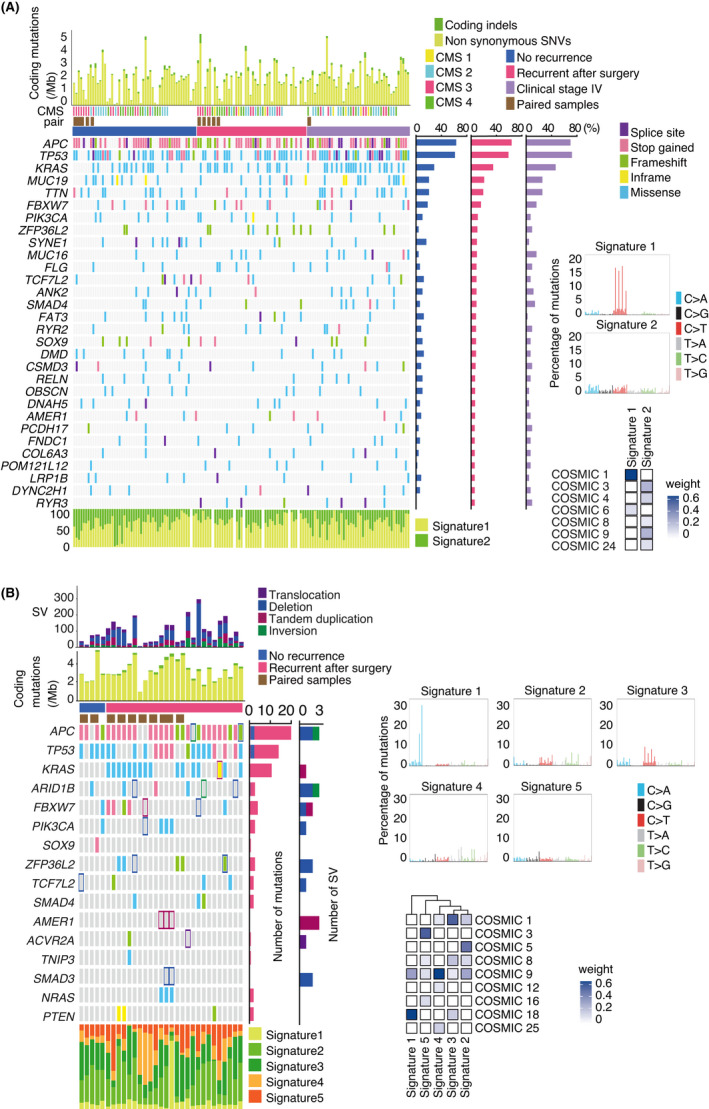
Mutational profiles in colorectal cancer. (A) Thirty most frequently mutated genes detected via whole‐exome sequencing and color‐coded mutation status for individual tumors. Mutation frequencies per gene in each study group are shown on the right. (B) Thirty most frequently mutated genes detected via whole‐genome sequencing and color‐coded mutation status for individual tumors. Structural variation frequencies in individual tumors and genes are shown at the top and on the right, respectively. Tumor pairs from the same patient are marked by brown squares. Frequencies of synonymous or non‐synonymous substitutions and insertions/deletions are shown at the top. Percentages of the mutational signatures are shown at the bottom. The cosine similarity of detected mutational signatures with COSMIC signatures is shown in the right panel

The results of target sequencing, WES, and WGS are shown in Figure 2 and Figure S1. The distribution of CMS categories was similar across patients with nrCRC, rCRC, and stage IV CRC. Our findings did not agree very well with a previous report of prognosis based on CMS classification in that the outcomes of patients with CMS4 tumors in this study were not particularly worse than those reported in the original report.^14^ The discrepancy might be due to ethnic differences or how the cohort in this study was selected from patients who were eligible for first‐line curable resection.

The detected single‐nucleotide variants were concordant with those reported in previous genomic studies of CRCs.^5^, ^6^ In our cohort, we did not detect any hypermutator (tumors having ≥10 mutations/Mb), microsatellite instability or mutation in *POLD1* and *POLE*, or germline mutation in *APC* and *MUTYH*. We identified somatic mutations in *APC* (target sequencing, 38/49 [77.6%]; WES, 90/141 [63.8%]; WGS, 27/31 [87.1%]), *TP53* (target sequencing, 29/49 [59.2%]; WES, 83/141 [58.9%]; WGS, 22/31 [71.0%]), *KRAS* (target sequencing, 18/49 [36.7%]; WES, 49/141 [34.8%]; WGS, 16/31 [51.6%]), and *PIK3CA* (target sequencing, 12/49 [24.5%]; WES, 15/141 [10.6%]; WGS, 4/31 [12.9%]). We also identified recurrent mutations in *ZFP36L2 (*WES, 14/141 [9.9%]; WGS, 5/31 [16.1%]) that were not included in the target sequence panel. Although mutations in *ZFP36L2* were not described in the first genomic analysis for CRC in the TCGA cohort,^5^ these are reportedly associated with metastatic CRC.^18^, ^19^

### 3.3 Copy number alterations in rCRC

**FIGURE 3:**
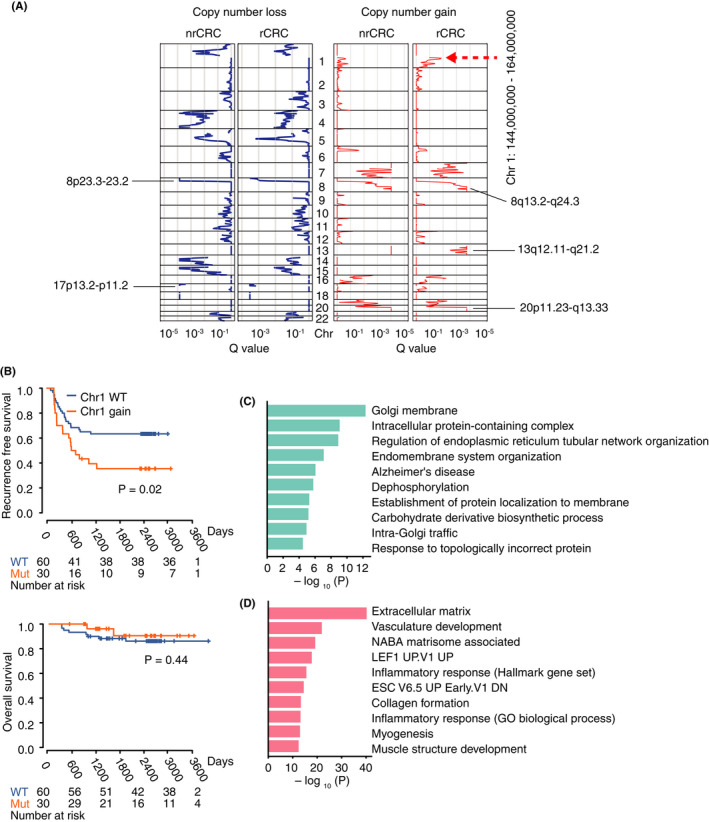
Copy number alterations in colorectal cancer. (A) Copy number alterations per subtype. Red and blue lines indicate Q‐values for gains and losses, respectively. (B) Overall survival and recurrence‐free survival according to the presence of indicated copy number alterations. Survival curves were estimated using the Kaplan–Meier method and compared using a two‐sided log‐rank test. (C) Pathways enriched in genes whose expression was upregulated in tumors with the copy number gain of chr 1: 144,000,000–164,000,000. (D) Pathways enriched in genes whose expression was downregulated in tumors with the copy number gain of chr 1: 144,000,000–164,000,000. CRC, colorectal cancer; nrCRC, non‐recurrent CRC; rCRC, recurrent CRC

On investigating the copy number alterations in nrCRCs and rCRCs, we noticed that copy number gains in chr 1: 144,000,000–164,000,000 were more frequent in nrCRCs (Figure 3A). Survival analysis indicated that CNAs in this genomic region may be prognostic factors (Figure 3B). Although there were several genes in this region related to colorectal cancer including *BCL9* ^20^ and *DDR2*,^21^, ^22^ we could not identify upregulated genes as a result of copy number gain in this region. Next, we analyzed the molecular pathways related to DEGs between cases with and without copy number gain in this region (Figures 3C,D) and found immune response to be negatively correlated with copy number gain in this region, and likely related to worse outcomes. By contrast, biological pathways associated with protein processing were upregulated in tumors with copy number gain in this region.

### 3.4 Structural variations (SVs) in rCRC

**FIGURE 4:**
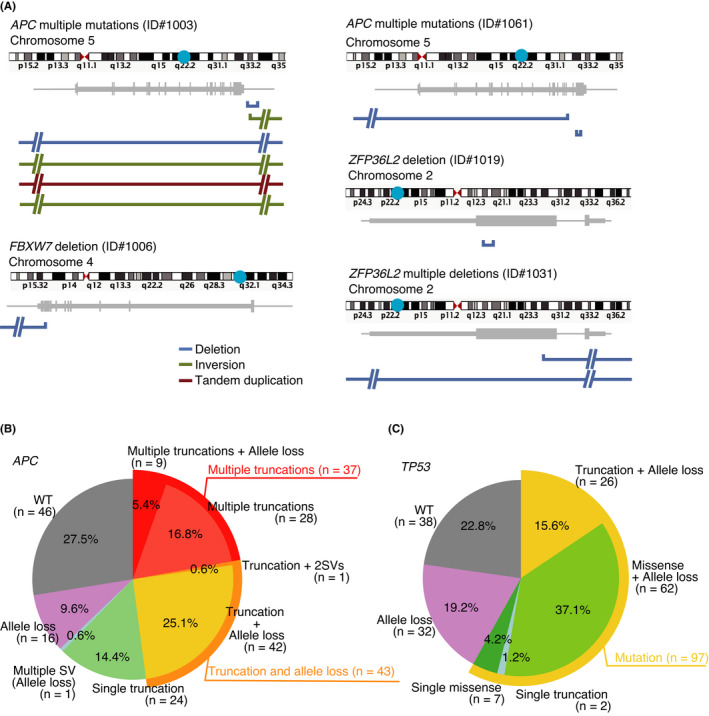
Structural variations in colorectal cancer. (A) Schematic diagrams of structural variations in representative genes. Chromosomes are shown at the top and genes are shown in the middle. In the lower part, deletions are shown in blue, inversions in green, and tandem duplications in red. (B and C) Biallelic mutational status of *APC* and *TP53*, respectively

Analysis of WGS data revealed a median of 76 (7–298) SVs, a fraction of which affected the major oncogenes (Figure 4A). SVs in *APC* were identified in two cases (deletion and inversion in tumor number 1003 and deletion in tumor number 1061); SV in *KRAS* was found in one case (tandem duplication in tumor number 1018); SVs in *FBXW7* were found in two cases (deletion in tumor number 1006 and tandem duplication in tumor number 1052) and SV in *PIK3CA* was found in one case (deletion in tumor number 1052).

In tumor number 1003, a large deletion was found in one allele of *APC*, resulting in loss of heterozygosity, whereas the other allele had multiple SVs, including a deletion in the exonic region. In tumor number 1061, a large deletion was found in one allele of *APC*, resulting in loss of heterozygosity, whereas the other allele had a disruptive frameshift deletion that probably eliminated *APC* function. In tumor number 1006, a deletion was found in the exon region of *FBXW7*. In tumor number 1052, a deletion was found in *PIK3CA* that resulted in the truncation of its transcript. In tumor number 1019, a deletion was found in the second exon of *ZFP36L2* that resulted in truncation, whereas in tumor number 1031, we detected a deletion encompassing the entire region of *ZFP36L2* and another in the promoter. Candidates for fusion genes were not identified in this study.

### 3.5 The substantial impact of SV detection on molecular diagnosis

Inactivation of *APC* occurs through sequential events, including point mutations, small insertions and deletions, and CNAs or SVs. Therefore, we investigated the combination of events that inactivate *APC*. Of 167 cases, 81 (48.5%) had biallelic *APC* inactivation, of which 37 (37/81, 45.7%) had biallelic point mutations and 43 (43/81, 53.1%) had point mutations accompanied by the loss of heterozygosity (Figure 4B). By contrast, *TP53* was more frequently altered by point mutations accompanied by loss of heterozygosity (88/97, 90.7%). In one tumor analyzed via WGS, two distinct SVs were found to affect the two *APC* alleles. Since a substantial number of SVs affecting major oncogenic events were not identified by WES, analysis of SVs via WGS was considered to facilitate the detection of oncogenic abnormalities that were not identified by target sequencing or WES.

### 3.6 Clinical significance of mutations in *ZFP36L2*

**FIGURE 5:**
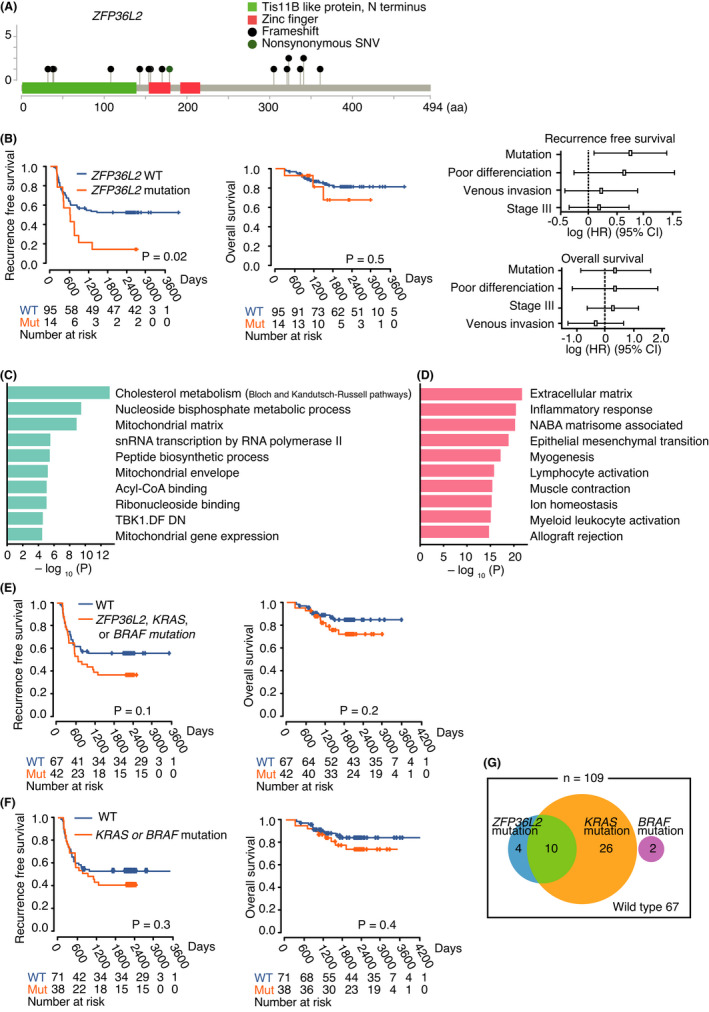
Clinical implication of mutations in *ZFP36L2*. (A) Mutations in *ZFP36L2* identified in this study. (B) Recurrence‐free survival and overall survival of patients with colorectal tumors according to the mutational status of *ZFP36L2*. Survival curves were estimated using the Kaplan–Meier method and compared using a two‐sided log‐rank test. Analysis of recurrence‐free survival and overall survival with key features using Cox proportional‐hazards model are also shown. (C) Pathways enriched in genes whose expression was upregulated in tumors with *ZFP36L2* mutations. (D) Pathways enriched in genes whose expression was downregulated in tumors with *ZFP36L2* mutations. (E) Recurrence‐free survival and overall survival of patients with colorectal tumors according to the mutational status of *ZFP36L2*, *KRAS*, or *BRAF*. (F) Recurrence‐free survival and overall survival of patients with colorectal tumors by mutational status of *KRAS* or *BRAF*. (G) Euler diagram showing the number of patients with mutations in *ZFP36L2*, *KRAS*, or *BRAF*. Survival curves were estimated using the Kaplan–Meier method and compared using a two‐sided log‐rank test

As described above (Figure 2), mutations and SVs in *ZFP36L2* were recurrently observed. We confirmed the sequencing results with manual inspection (Figure S2 and Table S1). Since a substantial fraction of mutations were frameshifts (Figure 5A), the detected mutations and SVs were considered to impair the function of *ZFP36L2* in most cases, suggesting *ZFP36L2* as a tumor suppressor in CRC. Since it appeared that mutations in *ZFP36L2* were enriched among rCRCs compared with among nrCRCs, we performed a survival analysis to explore the possibility that mutations in *ZFP36L2* were associated with tumor recurrence. Interestingly, RFS of patients with *ZFP36L2* mutations was significantly shorter than that of patients without the mutations (Figure 5B, Table S2). Non‐synonymous mutations in *ZFP36L2* were found in 30 of 594 cases from the TCGA cohort. Of the 223 cases with disease‐free survival data available, 10 had non‐synonymous mutations in *ZFP36L2*. However, no significant difference was detected in the disease‐free survival of patients with or without *ZFP36L2* mutations in the TCGA cohort (Figure S3).

DEGs detected on comparing tumors with or without *ZFP36L2* mutations were subjected to pathway analysis. Interestingly, tumors with *ZFP36L2* mutations had increased expression of genes associated with metabolic pathways such as cholesterol metabolism and mitochondrial function (Figure 5C). This finding suggests that tumors with *ZFP36L2* mutations might be characterized by a different metabolic state, making metabolic pathways a potential therapeutic target. However, further study is required. Expression of genes associated with immune response was suppressed in tumors with *ZFP36L2* mutations (Figure 5D), which might contribute to the poor outcome of patients with these tumors.

Although mutations in *KRAS* and *BRAF* are reportedly related to inferior treatment outcomes,^10^, ^11^ we did not observe significantly shorter survival in patients with *KRAS* or *BRAF* mutations, probably due to relatively small cohort size (Figure 5E,F). While 71% (10 of 14) of mutations in *ZFP36L2* were detected in patients with *KRAS* mutations (Figure 5G), inclusion of *ZFP36L2* mutations in patient stratification might increase the sensitivity of recurrence prediction with minimal loss of specificity (Table 2).

**TABLE 2:** Predictive values for mutations in marker genes

Marker genes	KRAS + BRAF	ZFP36L2	KRAS + BRAF + ZFP36L2
Recurrent			
Positive	23	12	27
Negative	34	45	30
Not recurrent			
Positive	15	2	15
Negative	37	50	37
Total	109	109	109
Sensitivity	0.404	0.211	0.474
Specificity	0.712	0.962	0.712
False negative rate	0.596	0.789	0.526
False positive rate	0.288	0.038	0.288
Positive predictive value	0.605	0.857	0.643
Negative predictive value	0.521	0.526	0.523

### 3.7 Clonal analysis

When tumors recurred after curative tumor resection, clinicians inferred their origin based on clinical information or pathological examination. Postoperative therapy is determined based on the tumor origin, i.e. whether cancer has emerged from the original tumor (“recurrence”) or de novo (“double cancer”). Thus, it would be important to know whether discrimination based on clinical and pathological information is correct.

In total, 31 tumors from 12 patients were considered to be recurrent or peritoneally disseminated and sharing the same origin as the primary tumors, whereas 23 tumors from 10 patients were considered to be double cancers. The assessments were performed independent of this study in the course of routine clinical practice, considering the tumor site, time intervals, and likelihood of tumor recurrence from a pathological viewpoint.

**FIGURE 6:**
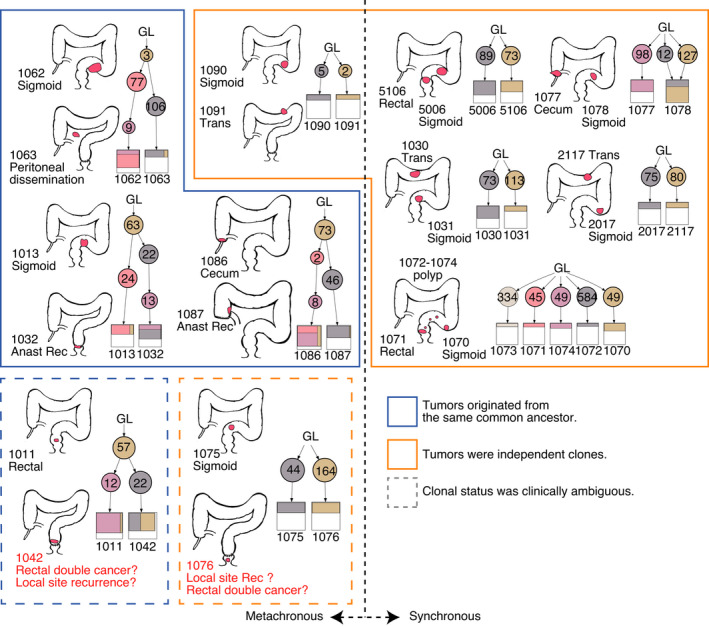
Clonal analysis of metachronous and synchronous tumors. Metachronous tumors are shown on the left side, and synchronous tumors are shown on the right side. For individual cases, the anatomical locations are shown on the left, and the clonal evolutionary process is shown on the right. Cases in which tumors were derived from the same clone are marked by a blue box and those derived from independent clones by an orange box. Cases where the origin of tumors was difficult to infer clinically are marked by dotted‐line boxes. Numbers in circles indicate the number of mutations considered for clonal analysis. Tumor identification is indicated by four‐digit numbers. Anast, anastomosis; GL, germ line; Rec, recurrence

Using clonal analysis of mutational information, we inferred that five cases of synchronous and two cases of metachronous cancer developed de novo, whereas four cases of metachronous cancer occurred via local metastasis or peritoneal dissemination (Figure 6). In all pairs of synchronous tumors, we found no shared mutations, including those in *APC*, *TP53*, or copy number alterations, indicating that the tumors may have initiated independently. By contrast, most metachronous lesions probably originated from a common ancestor as the initial sample and shared mutations in *APC* and *TP53*. In two cases, the clinical assessment was discordant with the clonal analysis. In the first case, the primary cancer (tumor number 1011) was a rectal adenocarcinoma, and rectal resection was performed as part of radical surgery. In the postoperative periodic examination, a new tumor was found on the oral side of the anastomosis of the former surgery (tumor number 1042). The tumor was not on the anastomosis site, and whether it was a local recurrence or double cancer was difficult to determine. Common mutations were detected, showing that they probably diverged from a common origin and were thus recurrent. In the second case, the primary lesion was sigmoid colon cancer (tumor number 1075). Sigmoidectomy was performed as radical surgery, and a new tumor (tumor number 1076) was detected on the anal side of the anastomosis during the routine postoperative examination; it was difficult to determine whether it was a local recurrence or double cancer. Rectal resection was performed, and pathological diagnosis revealed that the tumor showed no evidence of recurrent cancer. Clonal analysis indicated that there were no shared mutations, which strongly indicated that these emerged independently. Collectively, the analyses showed that clinical inference is primarily correct, and clonal analysis of cases with complex assessment may help improve clinical assessment.

## 4 DISCUSSION

Precise prediction of postoperative recurrence would help improve treatment strategies for stage II/III CRC. In this study, genomic analysis was conducted to identify predictive biomarkers for the detection of stage II/III CRC recurrence and to explore genomic abnormalities. For selected cases, we performed WGS to explore genomic abnormalities in more detail, and several new findings were obtained.

Our results showed that LOF mutations in *ZFP36L2* may be predictive of recurrence. *ZFP36L2* encodes a zinc‐finger RNA‐binding protein, which is reportedly involved in regulating mRNA stabilization^23^ and protein synthesis.^23^, ^24^ Previous studies have shown that ZFP36L2 suppresses the proliferation of colon cancer cell lines *in vitro* ^25^ and that LOF mutations in *ZFP36L2* are enriched in metastatic CRCs.^18^, ^19^ It was also reported that LOF of *ZFP36L2* promotes tumor progression by impairing apoptosis due to DNA damage.^26^ Therefore, *ZFP36L2* could be a tumor suppressor for CRC, and its deficiency may predict recurrence or worse outcomes. However, the findings were inconsistent with previously reported data that revealed the expression of *ZFP36L2* to be increased in 10% of gastric cancer, and ZFP36L2 to promote tumor proliferation.^27^ In addition, the analysis of publicly available TCGA data was not consistent with that of our cohort. Such discrepancies may be attributed to differences in ethnicity, sequencing procedures (including library preparation), or mutational analysis methods, which affect the detection sensitivity for *ZFP36L2* mutations. Indeed, mutations in *ZFP36L2* were not described in the original report of the TCGA cohort. Moreover, we found gene expression associated with the immune reaction to be altered in tumors with *ZFP36L2*, which might explain the mechanistic basis of recurrence. However, as the difference in analytical procedures may affect the detection of *ZFP36L2* mutations, a unified procedure along with a larger cohort would be required for elucidating the functions of *ZFP36L2*.

*ZFP36L2* mutation status was associated with differences in RFS, but OS did not differ by *ZFP36L2* mutation status; *ZFP36L2* mutation status may predict tumor recurrence, but not response to second‐line therapy. Further analysis is needed to clarify the prognostic impact of *ZFP36L2* mutation.

Our data showed that chromosomal SVs often involve major oncogenes related to CRC. For instance, we found both alleles of *APC* to be affected by SV in one case and a relatively large deletion leading to loss of heterozygosity in other cases. Such aberrations are likely to escape target sequencing or WES detection. Therefore, a comprehensive analysis via WGS and long‐read sequencing could help reveal genomic abnormalities and identify the underlying mechanism of action in CRC, such as mutations in *ZFP36L2*.

Clonal analysis was performed to examine the degree of concordance between clinical and genetic data. Our results showed that clinical assessment is precise, except for cases where the origin of tumors is difficult to infer based on clinical information alone. Clonal analysis based on sequencing data could contribute to improving clinical practice.

This study had the following limitations: (1) cases were selected based on the availability of tumor samples, and thus, comparisons between control groups were not possible; (2) our cohort was a mixture of patients with stage II and stage III CRC, and the influence of confounding factors was unavoidable. However, we selected the nrCRC group such that the percentage of stage II tumors would be the same in the rCRC and nrCRC groups, which, we believe, justifies our analysis. Further investigation will be required to validate the significance of *ZFP36L2* deficiency via prospective studies, especially for stage II CRC.

In conclusion, our study showed that detailed genomic analysis could improve the precise clinical characterization of CRC. In particular, *ZFP36L2* was identified as a candidate biomarker for rCRC. We also believe that our data will provide a clue to introduce WGS into clinical genomics in the future.

## CONFLICT OF INTEREST

All the authors declare that they have no competing interests.

## AUTHOR CONTRIBUTIONS

F.K., Y.T., Y.Y., S. Inoue, S.S. prepared and sequenced the sequencing libraries. F.K. and M.K. conducted data analysis. T.U. and S. K. processed and analyzed sequenced data. K.S. and S. Ishihara provided clinical specimens. S. Ishihara and H.M. conceived the study. M.K. wrote the manuscript with comments from H.M.

## ETHICS STATEMENT

All patients provided informed consent for the use of tumor specimens. The project was approved by the institutional ethics committees of The University of Tokyo (The Human Genome, Gene Analysis Research Ethics Committee; G10063, G10094, and G3546) and the National Cancer Center Research Institute (No. 2015–202), and was conducted in accordance with the guidelines specified in the Declaration of Helsinki.

## Supporting information


Table S1

Table S2

Figure S1
Figure S2
Figure S3
Figure S4

## Data Availability

Raw sequencing data were deposited in the Japanese Genotype‐phenotype Archive (http://trace.ddbj.nig.ac.jp/jga) hosted by the DNA Data Bank of Japan under the accession number JGAS00000000113 (NBDC number: hum0094).

## References

[1] Gill S , Loprinzi CL , Sargent DJ , et al. Pooled analysis of fluorouracil‐based adjuvant therapy for stage II and III colon cancer: who benefits and by how much? J Clin Oncol. 2004;22:1797‐1806. 15067028 10.1200/JCO.2004.09.059

[2] Figueredo A , Charette ML , Maroun J , Brouwers MC , Zuraw L . Adjuvant therapy for stage II colon cancer: a systematic review from the Cancer Care Ontario program in evidence‐based Care's gastrointestinal cancer disease site group. J Clin Oncol. 2004;22:3395‐3407. 15199087 10.1200/JCO.2004.03.087

[3] Kannarkatt J , Joseph J , Kurniali PC , al‐Janadi A , Hrinczenko B . Adjuvant chemotherapy for stage II colon cancer: a clinical dilemma. J Oncol Pract. 2017;13:233‐241. 28399381 10.1200/JOP.2016.017210

[4] Seshagiri S , Stawiski EW , Durinck S , et al. Recurrent R‐spondin fusions in colon cancer. Nature. 2012;488:660‐664. 22895193 10.1038/nature11282 PMC3690621

[5] The Cancer Genome Atlas Network . Comprehensive molecular characterization of human colon and rectal cancer. Nature. 2012;487:330‐337. 22810696 10.1038/nature11252 PMC3401966

[6] Yaeger R , Chatila WK , Lipsyc MD , et al. Clinical sequencing defines the genomic landscape of metastatic colorectal cancer. Cancer Cell. 2018;33:125‐136.e3. 29316426 10.1016/j.ccell.2017.12.004 PMC5765991

[7] Hu Z , Ding J , Ma Z , et al. Quantitative evidence for early metastatic seeding in colorectal cancer. Nat Genet. 2019;51:1113‐1122. 31209394 10.1038/s41588-019-0423-x PMC6982526

[8] Dalerba P , Sahoo D , Paik S , et al. CDX2 as a prognostic biomarker in stage II and stage III colon cancer. N Engl J Med. 2016;374:211‐222. 26789870 10.1056/NEJMoa1506597 PMC4784450

[9] Samowitz WS , Sweeney C , Herrick J , et al. Poor survival associated with the BRAF V600E mutation in microsatellite‐stable colon cancers. Cancer Res. 2005;65:6063‐6069. 16024606 10.1158/0008-5472.CAN-05-0404

[10] Taieb J , Zaanan A , le Malicot K , et al. Prognostic effect of BRAF and KRAS mutations in patients with stage III colon cancer treated with leucovorin, fluorouracil, and oxaliplatin with or without cetuximab. JAMA Oncol. 2016;2:643. 26768652 10.1001/jamaoncol.2015.5225

[11] Sinicrope FA , Shi Q , Smyrk TC , et al. Molecular markers identify subtypes of stage III colon cancer associated with patient outcomes. Gastroenterology. 2015;148:88‐99. 25305506 10.1053/j.gastro.2014.09.041 PMC4274188

[12] Kohsaka S , Tatsuno K , Ueno T , et al. Comprehensive assay for the molecular profiling of cancer by target enrichment from formalin‐fixed paraffin‐embedded specimens. Cancer Sci. 2019;110:1464‐1479. 30737998 10.1111/cas.13968 PMC6447855

[13] Shen R , Seshan VE . FACETS: allele‐specific copy number and clonal heterogeneity analysis tool for high‐throughput DNA sequencing. Nucleic Acids Res. 2016;44:e131. 27270079 10.1093/nar/gkw520 PMC5027494

[14] Guinney J , Dienstmann R , Wang X , et al. The consensus molecular subtypes of colorectal cancer. Nat Med. 2015;21:1350‐1356. 26457759 10.1038/nm.3967 PMC4636487

[15] Zhou Y , Zhou B , Pache L , et al. Metascape provides a biologist‐oriented resource for the analysis of systems‐level datasets. Nat Commun. 2019;10:1523. 30944313 10.1038/s41467-019-09234-6 PMC6447622

[16] Roth A , Khattra J , Yap D , et al. PyClone: statistical inference of clonal population structure in cancer. Nat Methods. 2014;11:396‐398. 24633410 10.1038/nmeth.2883 PMC4864026

[17] Popic V , Salari R , Hajirasouliha I , Kashef‐Haghighi D , West RB , Batzoglou S . Fast and scalable inference of multi‐sample cancer lineages. Genome Biol. 2015;16:1‐17. 25944252 10.1186/s13059-015-0647-8 PMC4501097

[18] Chen HN , Shu Y , Liao F , et al. Genomic evolution and diverse models of systemic metastases in colorectal cancer. Gut. 2022;71:322‐332. 33632712 10.1136/gutjnl-2020-323703 PMC8762014

[19] Mendelaar PAJ , Smid M , van Riet J , et al. Whole genome sequencing of metastatic colorectal cancer reveals prior treatment effects and specific metastasis features. Nat Commun. 2021;12:574. 33495476 10.1038/s41467-020-20887-6 PMC7835235

[20] Mani M , Carrasco DE , Zhang Y , et al. BCL9 promotes tumor progression by conferring enhanced proliferative, metastatic, and angiogenic properties to cancer cells. Cancer Res. 2009;69:7577‐7586. 19738061 10.1158/0008-5472.CAN-09-0773 PMC4321734

[21] Gao Y , Zhou J , Li J . Discoidin domain receptors orchestrate cancer progression: a focus on cancer therapies. Cancer Sci. 2021;112:962‐969. 33377205 10.1111/cas.14789 PMC7935774

[22] Sasaki S , Ueda M , Iguchi T , et al. DDR2 expression is associated with a high frequency of peritoneal dissemination and poor prognosis in colorectal cancer. Anticancer Res. 2017;37:2587‐2591. 28476831 10.21873/anticanres.11603

[23] Adachi S , Homoto M , Tanaka R , et al. ZFP36L1 and ZFP36L2 control LDLR mRNA stability via the ERK‐RSK pathway. Nucleic Acids Res. 2014;42:10037‐10049. 25106868 10.1093/nar/gku652 PMC4150769

[24] Hudson BP , Martinez‐Yamout MA , Dyson HJ , Wright PE . Recognition of the mRNA AU‐rich element by the zinc finger domain of TIS11d. Nat Struct Mol Biol. 2004;11:257‐264. 14981510 10.1038/nsmb738

[25] Suk F‐M , Chang C‐C , Lin R‐J , et al. ZFP36L1 and ZFP36L2 inhibit cell proliferation in a cyclin D‐dependent and p53‐independent manner. Sci Rep. 2018;8:2742. 29426877 10.1038/s41598-018-21160-z PMC5807420

[26] Noguchi A , Adachi S , Yokota N , et al. ZFP36L2 is a cell cycle‐regulated CCCH protein necessary for DNA lesion‐induced S‐phase arrest. Biol Open. 2018;7:bio031575. 29449217 10.1242/bio.031575 PMC5898266

[27] Xing R , Zhou Y , Yu J , et al. Whole‐genome sequencing reveals novel tandem‐duplication hotspots and a prognostic mutational signature in gastric cancer. Nat Commun. 2019;10:2037. 31048690 10.1038/s41467-019-09644-6 PMC6497673

