# Supplementary material for: Exploration of predictive biomarkers for postoperative recurrence of stage II/III colorectal cancer using genomic sequencing
Source: Cancer Med. 2022 Mar 28;11(18):3457–70. doi: 10.1002/cam4.4710 (PMC9487878; doi:10.1002/cam4.4710)
Supplement: Supplementary file 3 — Figure S1 Figure S2 Figure S3 Figure S4 [file CAM4-11-3457-s003.docx]

**Supplementary Figure S1**


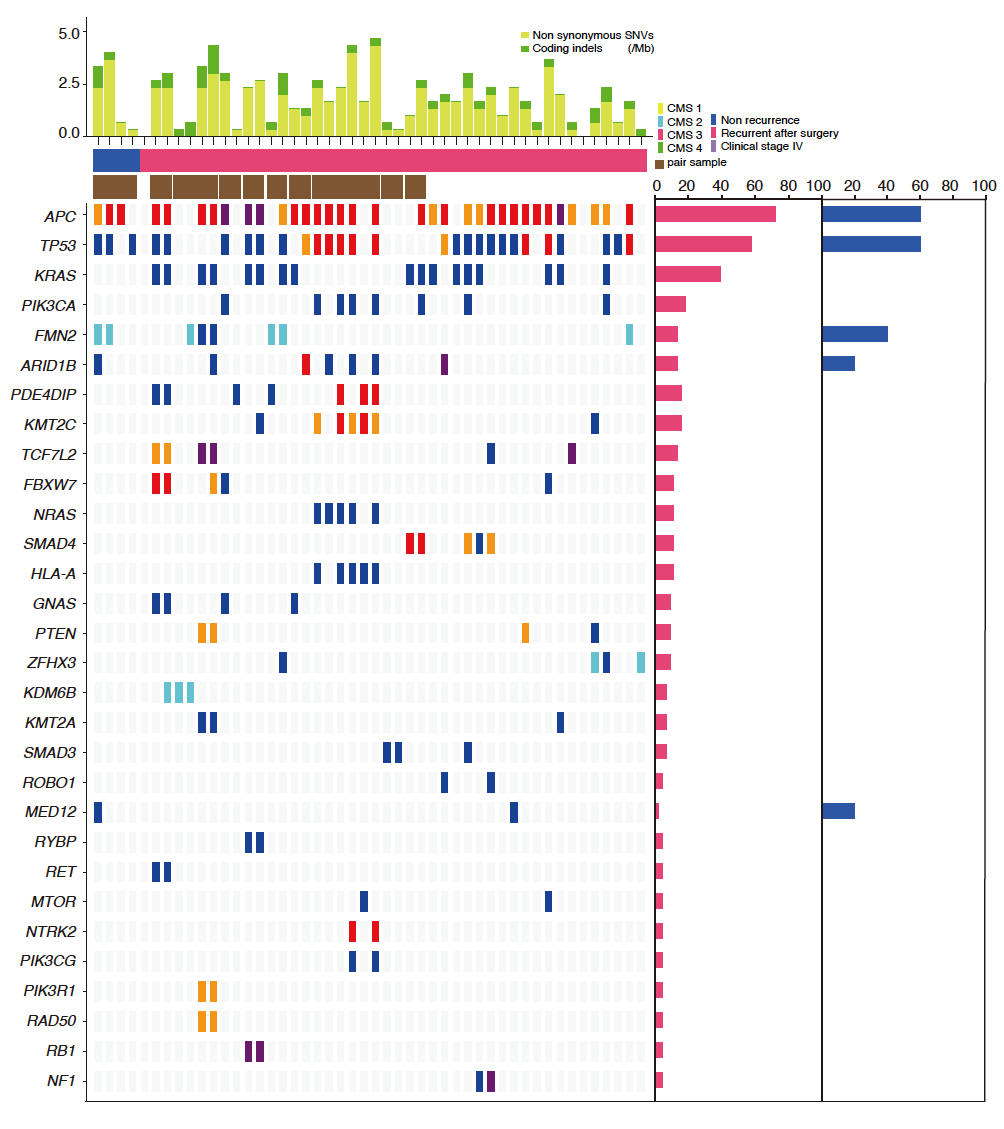


**Supplementary Figure S1 Mutational profiles in colorectal cancer**

Thirty most frequently mutated genes detected via target sequencing and color-coded mutation status for individual tumors. Mutation frequencies per gene in each study group are shown on the right. Tumor pairs from the same patient are marked by brown squares. Frequencies of synonymous or non-synonymous substitutions and insertions/deletions are shown at the top.

**Supplementary Figure S2**


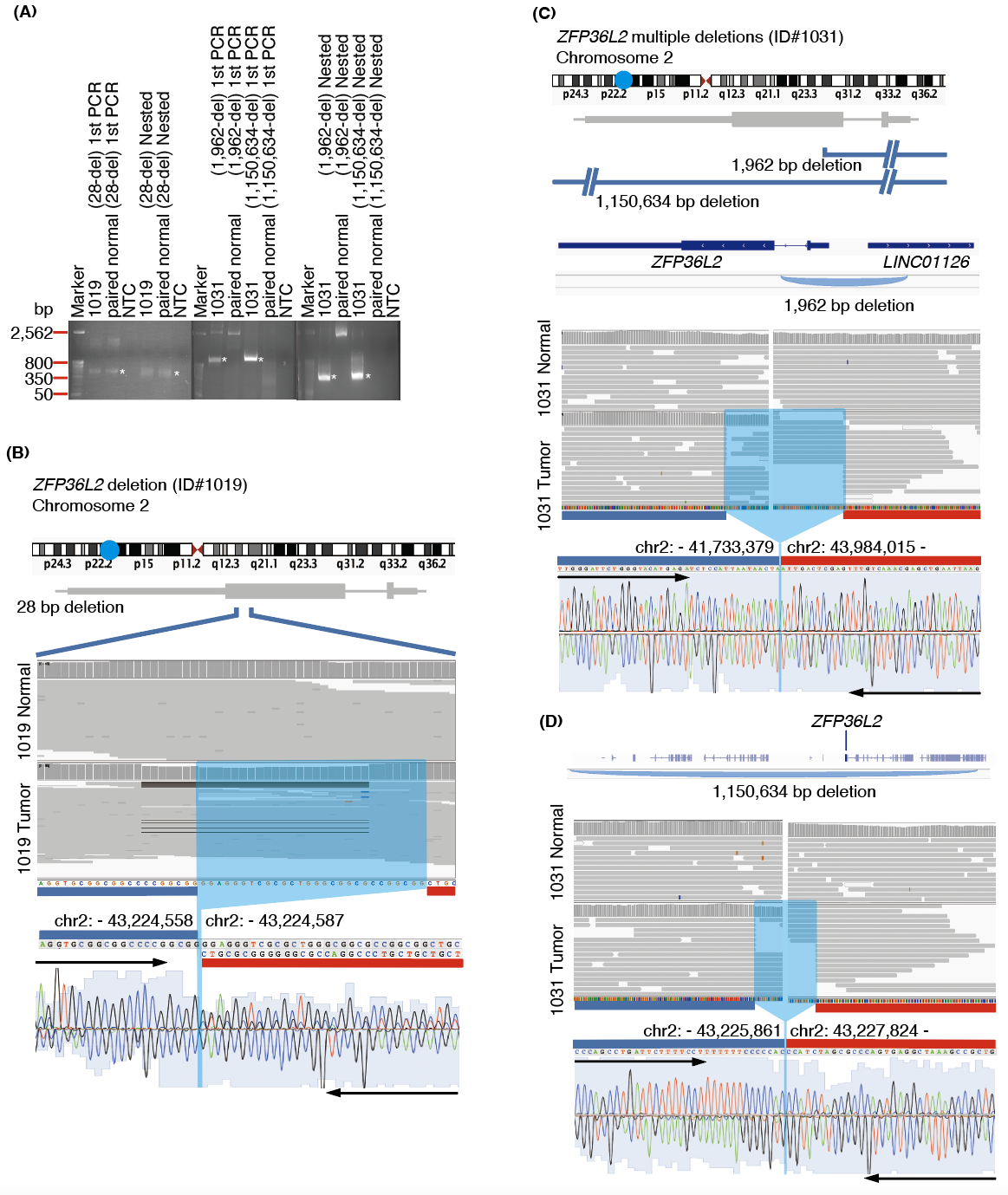


**Supplementary Figure S2 Validation of structural variations in *ZFP36L2***

**A**, Breakpoints associated with deletions in *ZFP36L2* were amplified by PCR of genomic DNA from patients ID#1019 and ID#1031. PCR amplicons with expected size are indicated with asterisks. **B**–**D**, PCR amplicons were subjected to Sanger sequencing analysis. The structures of the deletions are schematically shown with red and blue bars along with the results of read alignment shown in IGV genome viewer. The electrophoretograms of the sequencing results are shown with arrows indicating the direction of sequencing.

**Supplementary Figure S3**

**
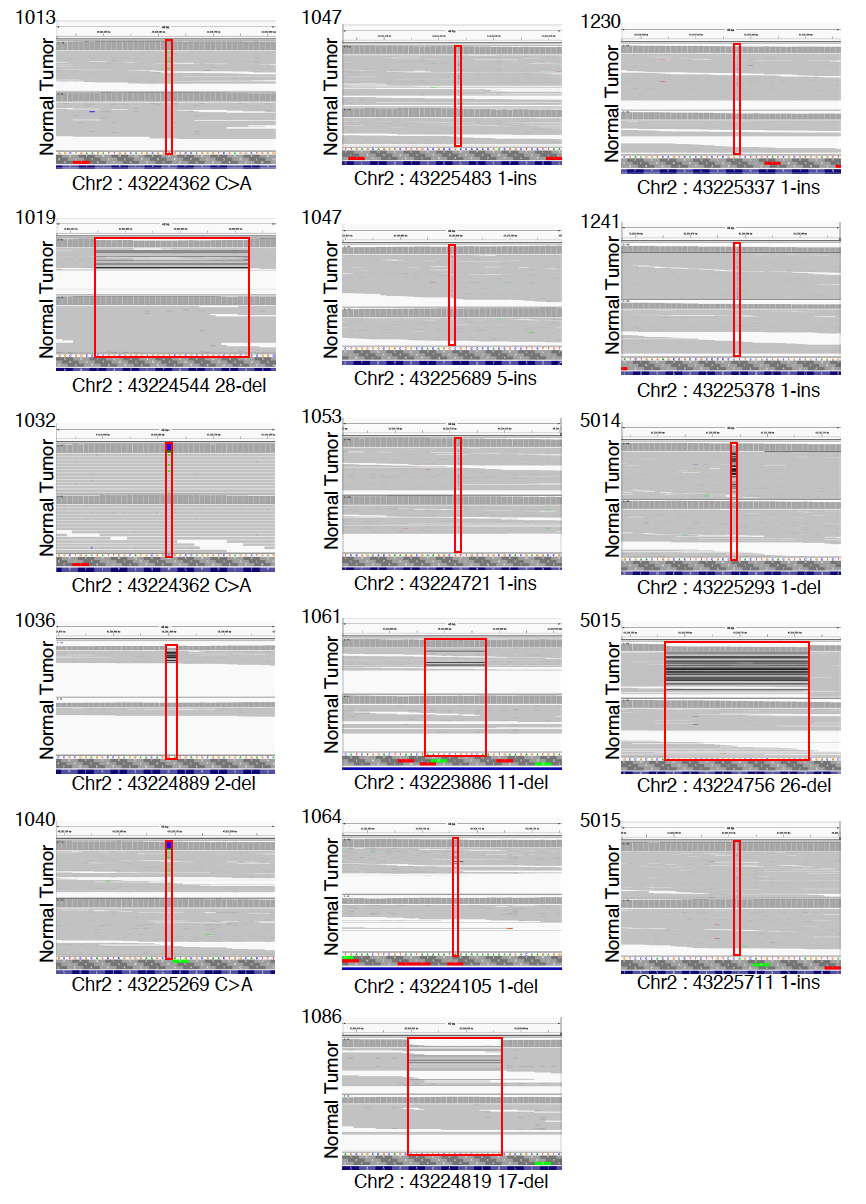
**

**Supplementary Figure S3 Screenshot images of sequencing reads for mutations in *ZFP36L2***

Bam files for sequencing reads that span the mutations in ZFP36L2 were visualized with IGV genome viewer. Reads from tumor samples and adjacent normal tissues are shown.

**Supplementary Figure S4**


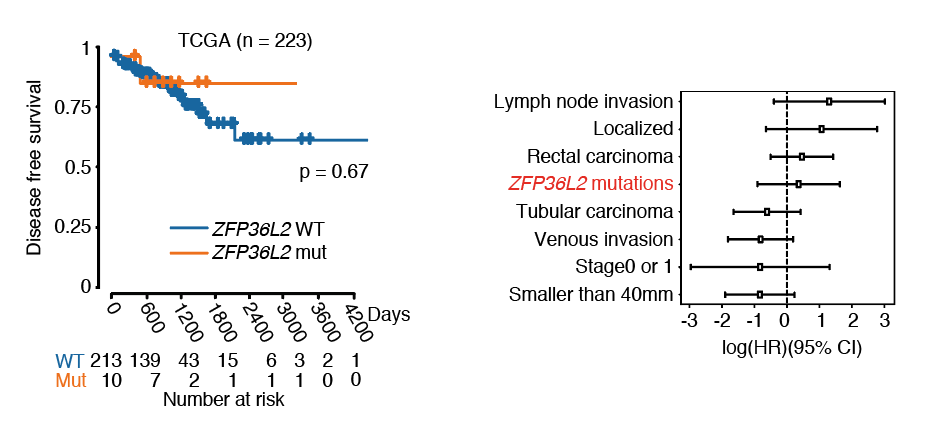


**Supplementary Figure S4 Clinical implication of mutations in *ZFP36L2* in TCGA cohort**

Disease -free survival of patients with colorectal tumors in TCGA cohort according to the mutational status of *ZFP36L2*. Survival curves were estimated using the Kaplan-Meier method and compared using a two-sided log-rank test. Analysis with key features using Cox proportional-hazards model were also shown.
